# Inhibition of synaptic transmission by anandamide precursor 20:4-NAPE is mediated by TRPV1 receptors under inflammatory conditions

**DOI:** 10.3389/fnmol.2023.1188503

**Published:** 2023-06-22

**Authors:** Diana Spicarova, Vladimir Nerandzic, David Muzik, Monica Pontearso, Anirban Bhattacharyya, Istvan Nagy, Jiri Palecek

**Affiliations:** ^1^Laboratory of Pain Research, Institute of Physiology of the Czech Academy of Sciences, Prague, Czechia; ^2^Section of Anaesthetics, Pain Medicine and Intensive Care, Department of Surgery and Cancer, Imperial College London, Faculty of Medicine, Chelsea and Westminster Hospital, London, United Kingdom; ^3^Department of Physiology, University of Debrecen, Debrecen, Hungary

**Keywords:** 20:4-NAPE, TRPV1, CB_1_, anandamide, NAPE-PLD, spinal cord, inflammation

## Abstract

Transient receptor potential ion channel, vanilloid subfamily, type 1 (TRPV1) cation channel, and cannabinoid receptor 1 (CB_1_) are essential in the modulation of nociceptive signaling in the spinal cord dorsal horn that underlies different pathological pain states. TRPV1 and CB_1_ receptors share the endogenous agonist anandamide (AEA), produced from N-arachidonoylphosphatidylethanolamine (20:4-NAPE). We investigated the effect of the anandamide precursor 20:4-NAPE on synaptic activity in naive and inflammatory conditions. Patch-clamp recordings of miniature excitatory postsynaptic currents (mEPSCs) from superficial dorsal horn neurons in rat acute spinal cord slices were used. Peripheral inflammation was induced by subcutaneous injection of carrageenan. Under naive conditions, mEPSCs frequency (0.96 ± 0.11 Hz) was significantly decreased after 20 μM 20:4-NAPE application (55.3 ± 7.4%). This 20:4-NAPE-induced inhibition was blocked by anandamide-synthesizing enzyme N-acyl phosphatidylethanolamine phospholipase D (NAPE-PLD) inhibitor LEI-401. In addition, the inhibition was prevented by the CB_1_ receptor antagonist PF 514273 (0.2 μM) but not by the TRPV1 receptor antagonist SB 366791 (10 μM). Under inflammatory conditions, 20:4-NAPE (20 μM) also exhibited a significant inhibitory effect (74.5 ± 8.9%) on the mEPSCs frequency that was prevented by the TRPV1 receptor antagonist SB 366791 but not by PF 514273 application. Our results show that 20:4-NAPE application has a significant modulatory effect on spinal cord nociceptive signaling that is mediated by both TRPV1 and CB_1_ presynaptic receptors, whereas peripheral inflammation changes the underlying mechanism. The switch between TRPV1 and CB_1_ receptor activation by the AEA precursor 20:4-NAPE during inflammation may play an important role in nociceptive processing, hence the development of pathological pain.

## 1. Introduction

The modulation of nociceptive synaptic transmission in the spinal cord dorsal horn underlies pathological pain states (Spicarova and Palecek, 2008, 2009; Spicarova et al., 2011; Li et al., 2015; Adamek et al., 2019; Heles et al., 2021; Mrozkova et al., 2021). Among others, two receptors - the Ca^2+^ permeable transient receptor potential ion channel, vanilloid subfamily, type 1 (TRPV1) cation channel and the cannabinoid receptor 1 (CB_1_) have been implicated to play important modulatory roles in spinal nociceptive signaling (Katona and Freund, 2008; Spicarova and Palecek, 2009; Spicarova et al., 2011). Interestingly, both TRPV1 and the CB_1_ receptors are activated by anandamide (AEA), which is produced from N-arachidonoylphosphatidylethanolamine (20:4-NAPE) in enzyme preparations (Wang et al., 2006), dorsal root ganglion (DRG) cultures (Varga et al., 2014) as well as spinal cord slices (Nerandzic et al., 2018).

TRPV1 receptors in the spinal cord are expressed predominantly on the central branches of small- and medium-sized DRG neurons, mainly in laminae I and II (Tominaga et al., 1998). Activation of those spinal TRPV1 receptors leads to increased glutamate release thus, increased frequency of postsynaptic excitatory currents on second-order spinal nociceptive neurons (Baccei et al., 2003; Spicarova et al., 2014b). The CB_1_ receptor, in addition to terminals of some central neurons and astrocytes, is also expressed in the terminals of small- and medium-sized DRG neurons in the superficial spinal cord (Ahluwalia et al., 2000; Farquhar-Smith et al., 2000; Hegyi et al., 2009; Veress et al., 2013). Notably, the CB_1_ receptor exhibits a high degree of co-expression with TRPV1 in DRG neurons (Ahluwalia et al., 2000; Binzen et al., 2006). Activation of the CB_1_ receptor inhibits transmitter release, including that due to TRPV1 activation in DRG neurons (Ahluwalia et al., 2003). In addition to reducing voltage-gated Ca^2+^ channel activity, this latter effect is due to inhibiting TRPV1 activity (Mahmud et al., 2009; Santha et al., 2010; Goncalves Dos Santos et al., 2020) but see Chen et al. (2016). Accordingly, CB_1_ receptor activation has been reported to suppress nociceptive behavior both in pathological pain states and in healthy organisms (Pertwee, 2009).

Various metabolic pathways could synthesize anandamide in DRG and spinal cord neurons, including via the Ca^2+^-sensitive N-acyl phosphatidylethanolamine phospholipase D (NAPE-PLD) and several Ca^2+^-insensitive synthetic pathways (van der Stelt et al., 2005; Vellani et al., 2008; Wang and Ueda, 2009; Snider et al., 2010; Varga et al., 2014). Expression of NAPE-PLD has been shown in DRG and spinal cord neurons and spinal glial cells (Nagy B. et al., 2009; Hegyi et al., 2012; Sousa-Valente et al., 2017). Importantly, in DRG neurons, NAPE-PLD is co-expressed with TRPV1 and CB_1_ receptors (Nagy B. et al., 2009; Sousa-Valente et al., 2017). Hence, in addition to being a retrograde signaling molecule (Katona and Freund, 2008), AEA also appears to act, at least in a major proportion of nociceptive DRG neurons, as an autocrine signaling molecule (Sousa-Valente et al., 2014, 2017).

Inflammation of peripheral tissues induces complex changes in the autocrine signaling system: it sensitizes spinal TRPV1 (Spicarova and Palecek, 2009) and up-regulates TRPV1 and CB_1_ receptor expression, whereas it reduces NAPE expression in DRG neurons (Amaya et al., 2006; Yu et al., 2008; Sousa-Valente et al., 2017). However, the CB_1_ receptor appears to get down-regulated in the spinal cord during joint inflammation (La Porta et al., 2013). Recently, we have reported that inflammation may modify the function of that autocrine signaling system as local AEA production induced by 20:4-NAPE application has different effects on spontaneous (s) and dorsal root stimulation evoked (e) EPSCs in second-order spinal neurons in naive and inflamed conditions. Although 20:4-NAPE strongly inhibited both sEPSCs frequency and eEPSCs amplitude both in naive and inflammatory conditions, the inhibitory effect was mediated through the CB_1_ receptors and through the CB_1_ receptor as well as TRPV1 in naive and inflammatory conditions, respectively (Nerandzic et al., 2018). However, it must be noted that sEPSC in the recorded neurons could be due to transmitter release following spontaneous action potential generation in excitatory or inhibitory spinal dorsal horn neurons as well as terminals of primary sensory neurons or descending nerve fibers. Therefore, it is important to exclude the effects of those spontaneous action potentials.

Here, to improve our understanding of the inflammation-induced alterations in the function of the AEA-TRPV1-CB_1_ receptor autocrine signaling circuitry in spinal cord primary afferents, we assessed the effects of local AEA synthesis from 20:4-NAPE on miniature (m) EPSCs recorded from superficial dorsal horn neurons under naive conditions and in a model of peripheral inflammation induced by carrageenan.

## 2. Materials and methods

### 2.1. Animals

Altogether, 29 male Wistar rats (Institute of Physiology CAS, Czech Republic) of postnatal age 19–23 days were randomly assigned to two groups: control (naive rats, *n* = 15) and inflamed (injected with carrageenan, *n* = 12). Animals were maintained under a temperature of 22 ± 2°C and light-controlled 12 h light/dark cycle conditions with free access to food and water. All experiments were approved by the local Institutional Animal Care and Use Committee and were carried out in accordance with the EU directive 2010/63/EU for animal experiments, the U.S. National Institutes of Health Guide for the Care and Use of Laboratory Animals, and guidelines of the International Association for the Study of Pain.

### 2.2. Spinal cord slice preparation

Male Wistar rats of postnatal days P19-P23 were used for spinal cord slice preparation, similar to previous experiments (Spicarova and Palecek, 2009). After anesthesia with ketamine (150 mg/kg, i.p.) and xylazine (16 mg/kg, i.p.) or 3% isoflurane, the lumbar spinal cord was removed and immersed in oxygenated ice-cold dissection solution containing (in mM) 95 NaCl, 1.8 KCl, 7 MgSO_4_, 0.5 CaCl_2_, 1.2 KH_2_PO_4_, 26 NaHCO_3_, 25 D-glucose and 50 sucrose. Animals were euthanized by subsequent medulla interruption and exsanguination. The spinal cord was fixed to a vibratome stage (VT 1000S; Leica, Germany) using cyanoacrylate glue in a groove between two agar blocks. Acute 300 μm thick transverse slices were cut, incubated in the dissection solution for 30 min at 33°C, stored in a recording solution at room temperature (21–24°C), and allowed to recover for 1 h before the electrophysiological experiments. The recording solution contained (in mM) 127 NaCl, 1.8 KCl, 1.2 KH_2_PO_4_, 2.4 CaCl_2_, 1.3 MgSO_4_, 26 NaHCO_3_, and 25 D-glucose. For the actual measurement, slices were transferred into a recording chamber perfused continuously with the recording solution at room temperature at a rate of ∼2 mL/min. All extracellular solutions were saturated with carbogen (95% O_2,_ 5% CO_2_) during the whole process.

### 2.3. Patch-clamp recording

Individual dorsal horn neurons were visualized using a differential interference contrast microscope (DM LFSA; Leica, Germany) equipped with a 63 × 0.90 water-immersion objective and an infrared-sensitive camera (KP-200P; Hitachi, Tokyo, Japan) with a standard TV/video monitor (VM-172; Hitachi, Tokyo, Japan). Patch pipettes were pulled from borosilicate glass tubing. When filled with intracellular solution, they had resistances of 3.5–6.0 MΩ. The intracellular pipette solution contained (in mM) 125 gluconic acid lactone, 15 CsCl, 10 EGTA, 10 HEPES, 1 CaCl_2_, 2 MgATP, and 0.5 NaGTP and was adjusted to pH 7.2 with CsOH. At room temperature, voltage-clamp recordings were performed in the whole-cell configuration with an AxoPatch 200B amplifier (Molecular Devices, Sunnyvale, CA, USA). Whole-cell responses were low-pass filtered at 2 kHz and digitally sampled at 10 kHz. The series resistance of the recorded neurons was routinely compensated by 80% and was monitored during the whole experiment. AMPA receptor-mediated mEPSCs were recorded from superficial dorsal horn neurons in laminae I and II_(outer)_, clamped at −70 mV in the presence of GABA_A_ receptor antagonist bicuculline (10 μM) and Glycine receptor antagonist strychnine (5 μM) to pharmacologically block the inhibitory synaptic transmission. Sodium channel blocker tetrodotoxin (TTX, 0.5 μM) was added to the recording solution to avoid action potential generation and distinguish mEPSC from spontaneous EPSC. The software package pCLAMP version 10.0 (Axon Instruments, Foster City, CA, USA) was used for data acquisition and subsequent offline analysis. Neurons with capsaicin-sensitive primary afferent input were identified by an increase in mEPSC frequency (>20%) following capsaicin (0.2 μM) application at the end of the experimental protocol.

### 2.4. Peripheral inflammation

Peripheral inflammation was induced in a group of animals (*n* = 12) 24 h before the preparation of the spinal cord slices. Both hind paws were injected subcutaneously under 3% isoflurane anesthesia by a 3% mixture of carrageenan (50 μL) in a physiological saline solution. Animals were left to recover in their home cages. This model of peripheral inflammation in rats replicates aspects of the human pain pathway. Naive animals were used as controls.

### 2.5. Behavioral testing

Animals used in the model of peripheral inflammation were tested for responsiveness to thermal stimuli before and 24 h after the carrageenan injection, when signs of peripheral inflammation (redness, hypersensitivity, and swelling) were present. Paw withdrawal latencies (PWLs) to radiant heat stimuli were determined for both hind paws using the Plantar Test apparatus (Ugo Basile, Gemonio, Italy). Rats were placed in non-binding, clear plastic cages on a glass plate and left to adapt for at least 20 min. The radiant heat was applied to the plantar surface of each hind paw until a deliberate escape movement of the paw was detected by the Plantar Test apparatus. The PWLs were tested 4 times for each hind paw with at least 5 min intervals between the trials. All procedures were based on our established behavioral testing (Pospisilova and Palecek, 2006; Uchytilova et al., 2021). Results from each hind paw were averaged. Baseline withdrawal latencies were determined in all animals before any experimental procedure. After behavioral testing, spinal cord slices were prepared and used for electrophysiological recordings.

### 2.6. Data and statistical analysis

For offline analysis of the recorded mEPSCs, data segments of 2-min duration were used for each experimental condition. Only mEPSCs with amplitudes 5 pA or greater (which corresponded to at least twice the noise level) were included in the frequency analysis. In the case of amplitude analysis, the same mEPSCs events were used. Data are expressed as means ± SEM, and a majority was normalized as a percentage of the control value (100%). Statistics were calculated using GraphPad Prism 9 Software. For statistical comparisons, non-parametric Wilcoxon signed rank test or repeated measures (RM) ANOVA on ranks were used where appropriate, and one-way ANOVA or Student’s *t*-test were used for data with normal distribution. Particular statistical tests used are indicated in the legend of figures or the results section. *p*-value <0.05 was considered statistically significant. Data from behavioral experiments were compared using paired *t*-test.

### 2.7. Materials

All chemicals used for extracellular and intracellular solutions were of analytical grade and purchased from Sigma Aldrich (St. Louis, MO, USA) and Tocris Bioscience (Bristol, UK). Capsaicin, SB 366791 - selectivity pA_2_ = 7.71 (Gunthorpe et al., 2004) - and PF 514273 - selectivity Ki = 1 nM (Dow et al., 2009) - were purchased from Tocris Bioscience and dissolved in DMSO. LEI-401 (Cayman Chemical, Michigan, USA) was also dissolved in DMSO, which always had a concentration of <0.1% in the final solution. 20:4-NAPE was obtained from Avanti Polar Lipids (Alabaster, AL, USA) dissolved in chloroform which had a concentration of <0.1% in the final solution. Drug concentrations were based on our previous studies (SB 366791, 10 μM; Spicarova and Palecek, 2009; Spicarova et al., 2014a; Nerandzic et al., 2018), preliminary experiments, and our previous studies (20:4-NAPE 20 μM and 200 μM; Nerandzic et al., 2018) or by considering Ki and the needed diffusion through the spinal cord slice (PF 514273, 0.2 μM; Nerandzic et al., 2018). Drugs were applied in the recording solution. Application of each drug lasted 4 min (20:4-NAPE, co-application of 20:4-NAPE + PF 514273, 20:4-NAPE + SB 366791, capsaicin) or 6 min for pretreatment (PF 514273, SB 366791). Carrageenan for induction of inflammation was purchased from Sigma Aldrich.

## 3. Results

### 3.1. Basic characterization of mEPSCs recorded from neurons in naive slices and after inflammation

Behavioral tests to thermal stimuli confirmed the development of peripheral inflammation in the experimental group of animals. The hind paw withdrawal responses significantly decreased 24 h after carrageenan injection to 8.39 ± 0.80 s from the control/pretreatment values 12.11 ± 1.4 s (*n* = 12, *p* < 0.01, paired *t*-test).

Altogether, recordings from 93 neurons located in the superficial dorsal horn [laminae I and II_(outer)_] were used for analysis in the electrophysiological experiments. The neurons recorded in the slices from naive animals had a mean basal mEPSC frequency of 0.96 ± 0.11 Hz (*n* = 60). The neurons recorded in slices from animals one day after the induction of the peripheral inflammation had a higher basal mEPSC frequency of 1.20 ± 0.13 Hz (*n* = 33) that was not statistically different from the naive control. Further, there was no significant difference in the mean mEPSC amplitudes during the application of the control solution (with TTX, bicuculline, and strychnine) recorded in the slices from naive animals (22.3 ± 1.0 pA, *n* = 60) and in slices from animals with inflammation (24.9 ± 1.4 pA, *n* = 33). Of the entire population of the 93 neurons included in this study, 88 (95%) were tested with capsaicin (0.2 μM) application at the end of the experiment and all of them showed an increase in mEPSC frequency, suggesting the presence of TRPV1 presynaptically.

### 3.2. The frequency of mEPSCs in dorsal horn neurons was decreased after the 20:4-NAPE application

**Figure 1:**
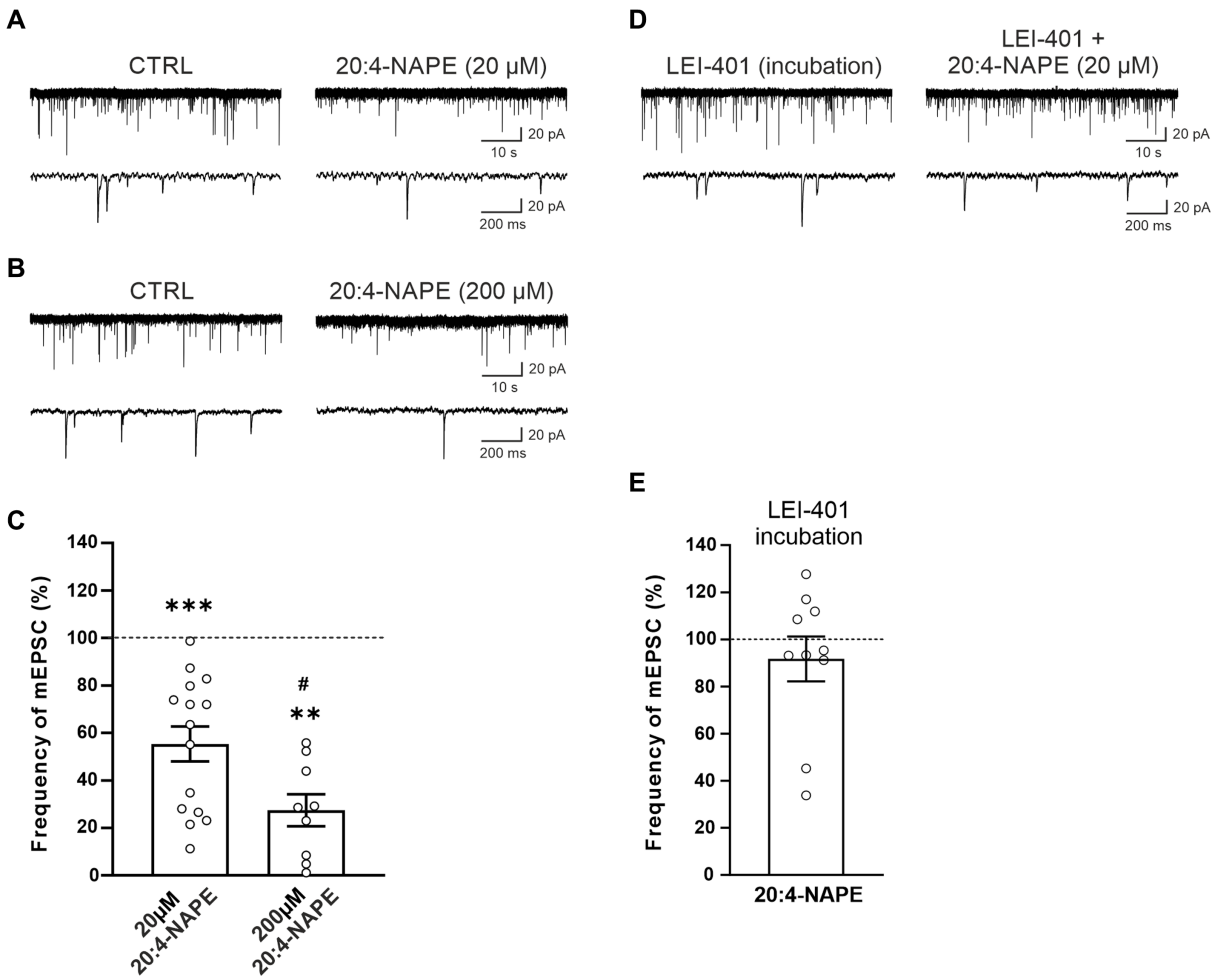
Application of anandamide precursor 20:4-NAPE induced robust inhibition of mEPSC frequency blocked by NAPE-PLD inhibitor in acute spinal cord slices from naive animals. An example of native recordings with 20:4-NAPE 20 μM **(A)** and 200 μM **(B)** application. **(C)** The decrease in mEPSC frequency after 20 μM concentration of 20:4-NAPE application was highly significant (*n* = 15, ***p < 0.001, Wilcoxon signed rank test), whereas 200 μM concentration induced a more pronounced decrease of mEPSC frequency (*n* = 9, **p < 0.01, Wilcoxon signed rank test; *versus control 100%, #*p* < 0.05, *t*-test, versus 20 μM 20:4-NAPE). **(D,E)** NAPE-PLD inhibitor LEI-401 (1 μM, at least 2 h) blocked the decrease of mEPSC frequency during the 20:4-NAPE (20 μM) application.

The effect of 20:4-NAPE application on mEPSCs recorded in the superficial dorsal horn neurons was tested with two different 20:4-NAPE concentrations (20 μM or 200 μM; 4 min application). The application of the lower concentration (20 μM), expressed as a percentage of the control value before the 20:4-NAPE administration, produced a robust decrease of the mEPSC frequency (55.3 ± 7.4%, *n* = 15, *p* < 0.001; Figures 1A,C). The amplitude of mEPSC during the application of 20:4-NAPE (20 μM) was not significantly changed from the control value (control: 29.2 ± 2.4 pA, 20:4-NAPE: 27.3 ± 2.8 pA, *n* = 15). The inhibitory effect of the higher 20:4-NAPE concentration on mEPSC frequency was even more pronounced (20:4-NAPE, 200 μM: 27.5 ± 6.8%, *n* = 9, *p* < 0.01; Figures 1B,C) reaching statistically significant difference also from the effect of 20 μM 20:4-NAPE (*p* < 0.05; Figure 1C). In addition, application of 200 μM 20:4-NAPE induced a significant decrease of the mEPSC amplitude (control: 21.7 ± 2.1 pA, 20:4-NAPE: 16.8 ± 1.9 pA, *n* = 9, *p* < 0.001, paired *t*-test). The effect of 200 μM 20:4-NAPE application on the amplitude indicates that in addition to modulating transmitter release, 20:4-NAPE may also significantly affect ion channels on the postsynaptic membrane. We aimed to study how 20:4-NAPE modulates TRPV1 and CB_1_ receptor on presynaptic endings in the dorsal horn. Therefore, in the rest of the study, we used the lower 20 μM concentration, which does not affect mEPSC amplitude.

We have shown previously that 20:4-NAPE application to spinal cord slices results in anandamide synthesis. NAPE-PLD has been suggested to be the main enzyme synthesizing anandamide from its precursor 20:4-NAPE. Therefore, we used the NAPE-PLD inhibitor LEI-401 in further experiments. Incubation of spinal cord slices in the bath containing LEI-401 (1 μM, at least 2 h) and subsequent acute application of 100 nM LEI-401 in the recording chamber blocked the 20:4-NAPE (20 μM, 4 min) mediated decrease of the mEPSC frequency (91.7 ± 9.5%, *n* = 10; Figures 1D,E). The amplitude of mEPSC during the application of 20:4-NAPE (20 μM, 4 min) in the presence of LEI-401 decreased significantly from the control value (control: 18.3 ± 2.1 pA, 20:4-NAPE: 14.9 ± 1.0 pA, *n* = 10). This finding indicated that the 20:4-NAPE-induced inhibitory effect was mediated through NAPE-PLD-synthesized anandamide.

### 3.3. Inhibition of mEPSCs frequency induced by 20:4-NAPE was prevented by CB_1_ antagonist but not by TRPV1 antagonist under the control conditions

**Figure 2:**
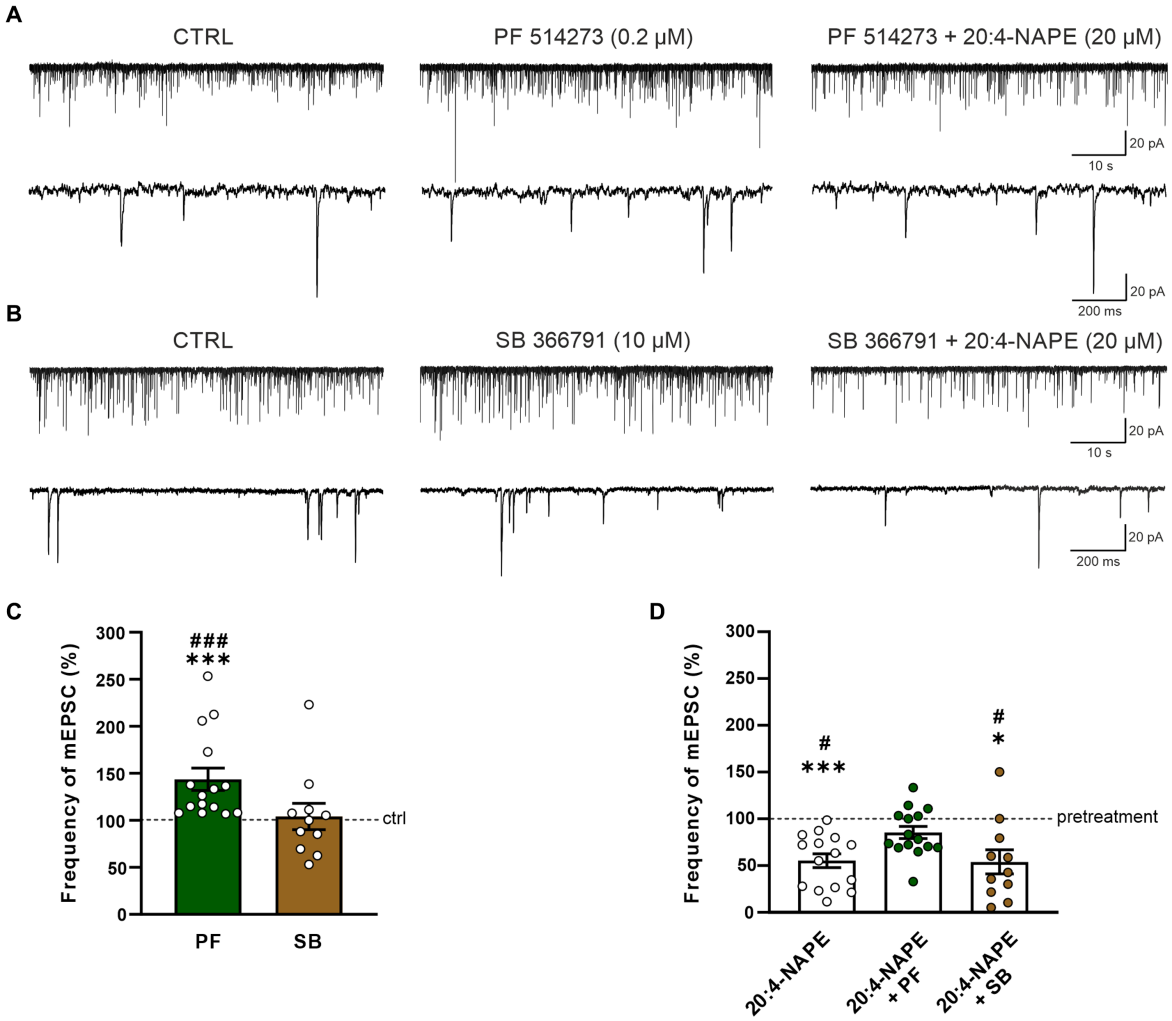
Effect of CB_1_ and TRPV1 antagonists application on 20:4-NAPE-induced inhibition of mEPSC frequency in naive slices. **(A,C)** The application of CB_1_ antagonist PF 514273 (0.2 μM) significantly increased the frequency of the recorded mEPSC, as is evident in the native recording example **(A)** and the averaged data graph (**C**, *n* = 15, ***p < 0.001 vs. control, ###*p* < 0.001 vs. SB 366791, one-way ANOVA on ranks, Kruskal-Wallis test, Dunn’s *post hoc* test). Subsequent co-application of PF 514273 (0.2 μM) and 20:4-NAPE (20 μM) did not change the mEPSC frequency compared to the antagonist pretreatment **(D)**. **(B,C)** TRPV1 antagonist SB 366791 application (10 μM) did not change the mEPSC frequency compared to the control level (*n* = 11). Subsequent application of 20:4-NAPE (20 μM) in the presence of SB 366791 (10 μM) induced significant inhibition of the mEPSC frequency **(B,D)**. **(D)** The summary graph shows a comparison of 20:4-NAPE-induced inhibition with the effects of 20:4-NAPE co-application with CB_1_ and TRPV1 antagonists. Data are expressed as a percentage of the pretreatment. PF 514273 prevented the 20:4-NAPE-induced inhibitory effect on mEPSC frequency, while SB 3667 did not (*p < 0.05, ***p < 0.001 vs. pretreatment (100%), Wilcoxon signed rank test; #*p* < 0.05 vs. co-application of PF 514273 + 20:4-NAPE, one-way ANOVA with Tukey *post hoc* test).

To find the contribution of the CB_1_ receptor, the selective CB_1_ antagonist PF 514273 (0.2 μM, 6 min) was applied before 20:4-NAPE application. The frequency of the mEPSC after the PF 514273 application increased significantly compared to the control value (143.7 *±* 11.9%, *n* = 15, *p* < 0.05; Figures 2A,C). During the subsequent co-application of 20:4-NAPE (20 μM) and PF 514273 (0.2 μM, 4 min), the mEPSC frequency remained increased when compared to the control basal frequency (121.3 ± 12.2%). In order to compare the overall effect of 20:4-NAPE under the different experimental conditions and to diminish any influences of the antagonist applications alone, data were also analyzed as a percentage of the previous condition (=100%), and the differences were statistically evaluated. Under this assessment, the mEPSC frequency after PF 514237 + 20:4-NAPE co-application as a percentage of the PF 514273 pretreatment did not change and was 85.3 ± 6.5% (Figure 2D). The amplitude of the mEPSCs remained unchanged during the entire recording (control: 23.3 ± 1.2 pA, PF 514273: 22.8 ± 1.4 pA, PF 514273 + 20:4-NAPE: 22.5 ± 1.0 pA, *n* = 15). 20:4-NAPE application did not inhibit the mEPSC frequency when CB_1_ was blocked in spinal cord slices.

The possible role of TRPV1 receptors in the 20:4-NAPE-induced inhibition was tested by application of the TRPV1 selective antagonist SB 366791 in the same way the CB_1_ antagonist was used. When SB 366791 (10 μM, 6 min) was applied, the mEPSC frequency did not change from the original control value (104.1 ± 14.0%, *n* = 11; Figures 2B,C). During the subsequent co-application of 20:4-NAPE (20 μM) and SB 366791 (10 μM, 4 min), the mEPSC frequency exhibited a significant reduction compared to SB 366791 pretreatment (53.9 ± 12.9%, *p* < 0.05; Figures 2B,D). This mEPSC frequency reduction was similar to the decrease induced by 20:4-NAPE application alone (Figures 1C, 2D). The TRPV1 antagonist application did not induce any significant changes in the mEPSC amplitudes (control: 23.1 ± 1.9 pA, SB 366791: 21.2 ± 1.8 pA, SB 366791 + 20:4-NAPE: 20.2 ± 1.5 pA, *n* = 11). These experiments suggested that under naive conditions, the inhibitory effect of 20:4-NAPE on the mEPSC frequency is mediated by CB_1_ receptors activation.

### 3.4. The inhibitory effect of 20:4-NAPE was prevented by TRPV1 antagonist but not by CB_1_ antagonist in a model of peripheral inflammation

**Figure 3:**
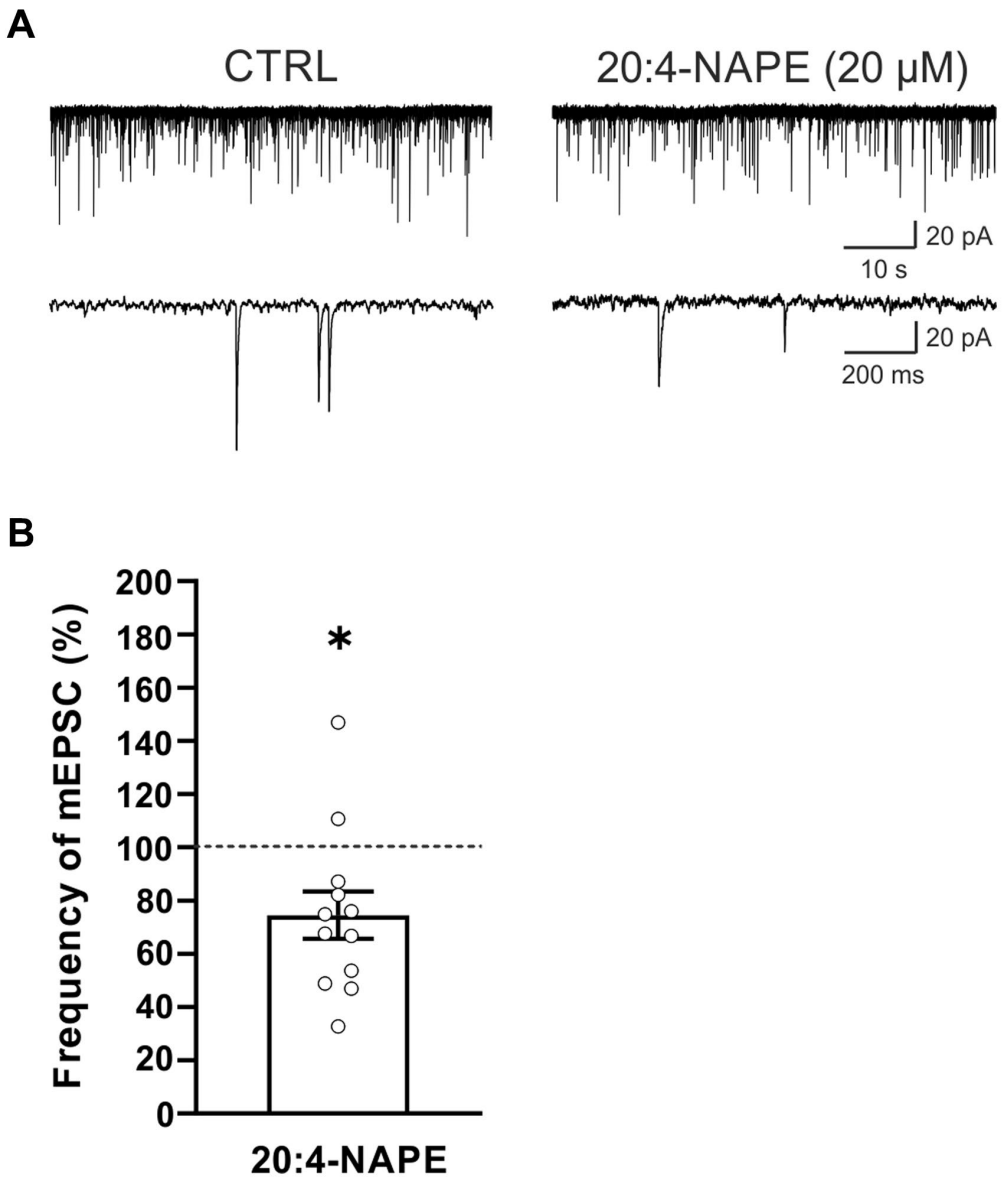
Application of 20:4-NAPE induced inhibition of mEPSC frequency in animals with peripheral inflammation. **(A)** Native recordings show inhibitory effect of 20:4-NAPE (20 μM) application on the mEPSC frequency in the dorsal horn 24 h after the induction of peripheral inflammation. **(B)** The average mEPSC frequency decreased during 20:4-NAPE (20 μM) application significantly (*n* = 12, *p < 0.05, Wilcoxon signed rank test).

To find the contribution of the TRPV1 receptor, spinal cord slices from rats with peripheral inflammation were used. Application of 20:4-NAPE (20 μM, 4 min) inhibited mEPSC frequency in 10 of the 12 recorded neurons (74.5 ± 8.9%, *n* = 12, *p* < 0.05; Figure 3). The inhibition was similar to that in naive conditions (Figure 1C). There was no statistically significant difference between 20:4-NAPE-induced inhibition in slices from naive and inflamed animals. The amplitude of mEPSC after the 20:4-NAPE application did not change (control: 26.5 ± 2.0 pA, 20:4-NAPE: 26.1 ± 2.2 pA, *n* = 12).

**Figure 4:**
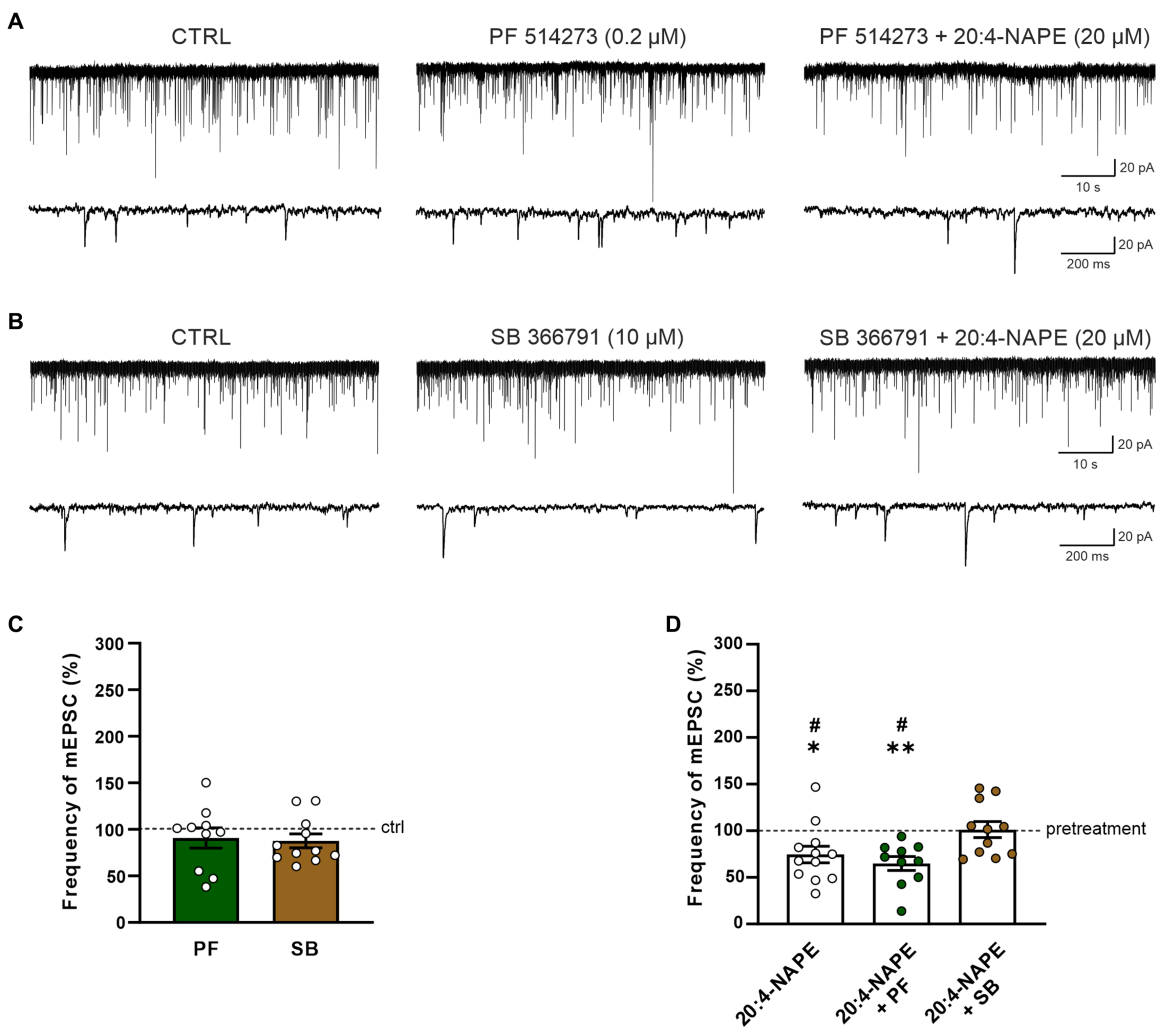
Effect of CB_1_ and TRPV1 antagonists application on 20:4-NAPE-induced inhibition of mEPSC frequency under inflammatory conditions. **(A,C)** CB_1_ antagonist PF 514273 (0.2 μM) application did not change the mEPSC frequency (*n* = 10). Subsequent co-application of 20:4-NAPE (20 μM) and PF 514273 (0.2 μM) induced strong inhibition **(D)**. **(B,C)** The frequency of mEPSC did not change during the application of TRPV1 antagonist SB 366791 (10 μM, *n* = 11). Subsequent co-application of 20:4-NAPE (20 μM) and SB 366791 (10 μM) also did not change the mEPSC frequency significantly (*n* = 11). **(D)** Data expressed as a percentage of pretreatment show that SB 366791 application (*n* = 11) prevented the 20:4-NAPE-induced (*n* = 12) inhibitory effect on mEPSC frequency under the inflammatory conditions, while PF 514273 did not (*n* = 10, *p < 0.05, **p < 0.01 versus pretreatment (100%) Wilcoxon signed rank test; ^#^*p* < 0.05 vs. co-application of SB 366791 + 20:4-NAPE, one-way ANOVA with Tukey *post hoc* test).

In the second set of experiments, the effect of CB_1_ receptor antagonist PF 514273 (0.2 μM, 6 min) was tested on 20:4-NAPE-induced inhibition of mEPSCs frequency under the inflammatory conditions. In contrast to the naive conditions, the antagonist alone did not evoke any significant change in the frequency of the mEPSCs (90.7 ± 10.9%, *n* = 10; Figures 4A,C). Subsequent 20:4-NAPE (20 μM) and PF 514273 (0.2 μM, 4 min) co-application elicited a significant decrease of the mEPSC frequency expressed as a percentage of PF 514273 pretreatment (64.9 ± 7.5%, *p* < 0.01; Figures 4A,D). The amplitude of the mEPSCs did not change significantly during the whole experiment (control: 26.1 ± 3.6 pA, PF 514273: 24.9 ± 3.1 pA, PF 514273 + 20:4-NAPE: 26.1 ± 3.3 pA, *n* = 10).

In the next set of experiments, the effect of the TRPV1 receptor antagonist was tested under the inflammatory conditions. First, the selective antagonist SB 366791 was applied alone (10 μM, 6 min), and then 20:4-NAPE (20 μM) was co-administered with it for 4 min. There was virtually no change in the mEPSC frequency during the SB 366791 pretreatment (87.7 ± 7.5%, *n* = 11; Figures 4B,C). In contrast to naive conditions, during the SB 3366791 and 20:4-NAPE co-application, the mean mEPSC frequency remained close to the control level (101.2 ± 8.7%, *n* = 11; Figures 4B,D) expressed as a percentage of the pretreatment. During both applications (SB 366791 or SB 366791 + 20:4-NAPE), the mEPSC amplitude was not changed compared to the basal level (control: 23.6 ± 2.5 pA, SB 366791: 21.0 ± 1.4 pA, SB 366791 + 20:4-NAPE: 20.8 ± 1.4 pA, *n* = 11). These results suggest that the 20:4-NAPE-induced inhibitory effect on the mEPSC frequency is preferentially mediated by TRPV1 receptors under the inflammatory conditions (Figure 4D).

## 4. Discussion

Recently, we have reported that application of 20:4-NAPE, a common substrate of all anandamide-synthesizing enzymatic pathways (Wang and Ueda, 2009; Snider et al., 2010), to rat spinal cord slices induces inhibitory effects on dorsal root stimulation-evoked and spontaneous EPSC (Nerandzic et al., 2018). While those inhibitory effects are mediated via the CB_1_ receptor in slices prepared from naive animals, they are mediated both through the CB_1_ receptor and TRPV1 in slices prepared from animals at one day after carrageenan injection into the paw. As spontaneous action potential generation in spinal dorsal horn circuitries can significantly contribute to the generation of sEPSC and eEPSC in dorsal horn neurons, here, using spinal cord slices of another set of naive and carrageenan injected rats, we studied the effect of 20:4-NAPE further, via assessing mEPSC frequency in superficial spinal dorsal horn neurons, both in naive condition and after inducing inflammation of peripheral tissues. Assessing mEPSCs in secondary sensory neurons of the superficial spinal dorsal horn, which constitute the first postsynaptic neurons in the nociceptive pathways, is important because it shows action potential-independent synaptic events. Further, here we also assessed the enzymatic pathway responsible for the 20:4-NAPE-evoked anandamide production in the spinal cord, and we report the involvement of NAPE-PLD.

Our results demonstrate that 20:4-NAPE application has a concentration-dependent inhibitory effect on mEPSC frequency hence directly on the spontaneous transmitter release from presynaptic terminals in the spinal dorsal horn. The TTX application excluded the involvement of spinal neuronal circuits in this process. We have recently reported similar 20:4-NAPE-produced inhibitory effects on sEPSC frequency (without TTX) and dorsal root stimulation-evoked EPSC amplitude in the spinal dorsal horn in both naive and inflammatory conditions (Nerandzic et al., 2018). In that study, 20 μM concentration of 20:4-NAPE induced the similar inhibitory effect on sEPSC, approximately 50% in naïve and inflammatory conditions. This finding strongly indicates that IC_50_ of 20:4-NAPE is the same in both examined conditions. The conversion of 20:4-NAPE to AEA in enzyme preparations, cultured primary sensory neurons, and spinal cord slices makes it highly likely that the effect is indirectly produced through *de novo* AEA synthesis (Wang and Ueda, 2009; Snider et al., 2010; Varga et al., 2014; Nerandzic et al., 2018). The conversion of 20:4-NAPE into AEA could be mediated by Ca^2+^-independent or/and Ca^2+^-dependent (NAPE-PLD) enzymatic pathways, many of which are expressed in various sub-populations of primary sensory neurons, as well as some neurons and glia cells in the spinal dorsal horn (Nagy B. et al., 2009; Hegyi et al., 2012; Varga et al., 2014; Sousa-Valente et al., 2017). Thus, the inhibitory effects on mEPCS frequency must have been due to an increase in AEA concentration by the activity of those AEA-synthesizing enzymes. Such synthesis leads to anandamide reaching sufficient concentration to produce effects in a limited number of cells. Based on the restricted movement of the lipophilic anandamide in the aqueous environment (Di Scala et al., 2018), we propose that anandamide that induced reduction in mEPSC frequency during 20 μM 20:4-NAPE application was synthesized in close proximity to downstream effectors (i.e., receptors and synaptic vesicles), hence in presynaptic terminals formed by primary sensory neurons. Our present experiments show the prevention of the 20:4-NAPE-induced inhibitory effect using NAPE-PLD inhibitor LEI-401 application, which indicates that the activity of NAPE-PLD mediated the effect - most likely via anandamide synthesis. In addition, the 20:4-NAPE-induced inhibitory effect we observed on mEPSC frequency indicates the presynaptic mechanism of action. We suggest that NAPE-PLD mainly localized presynaptically on the central endings of primary afferent neurons converted 20:4-NAPE to anandamide to target nearby receptors.

Interestingly, at 200 μM, 20:4-NAPE, in addition to reducing mEPCS frequency, also reduced mEPSC amplitude. The change of properties, type, or number of postsynaptic receptors typically leads to a change in the amplitude of mEPSC. This finding suggests that at 200 μM 20:4-NAPE, AEA from some source reached the recorded neurons in a concentration sufficient to produce a postsynaptic effect. Unexpectedly, during NAPE-PLD inhibition, the amplitude of miniature EPSC diminished after 20 μM 20:4-NAPE application, indicating possible postsynaptic effect of the 20:4-NAPE application on superficial dorsal horn neurons. Several anandamide synthesizing enzymes are expressed in the dorsal horn, and a competition of various pathways for the substrate caused by LEI-401 application could underlie this postsynaptic effect.

The CB_1_ receptor and TRPV1 constitute the primary targets for anandamide (Zygmunt et al., 1999; Ahluwalia et al., 2003). Therefore, we used antagonists of those two receptors to find the mechanism of the 20:4-NAPE-induced inhibitory effect. Application of the CB_1_ receptors antagonist alone induced a significant increase in the basal mEPSC frequency in neurons recorded in naive slices but not in slices prepared from carrageenan-injected animals. CB_1_ receptor activity has been suggested to attenuate nociceptive signaling by trimeric G_i/o_-protein cascade (Howlett et al., 2002). This cascade stimulates inwardly rectifying and A-type outward potassium channels and decreases the activity of high voltage-activated N- and P/Q-type Ca^2+^ channels (Pertwee, 2006), leading to glutamate release suppression from presynaptic endings. The observed increase in mEPSC frequency in naive slices was thus most likely due to the loss of CB_1_ receptor inhibitory activity in central terminals of primary afferent fibers (Nyilas et al., 2009). This CB_1_ receptor activation-dependent inhibition was hidden by spontaneous excitatory activity in local neuronal circuits during sEPSC recording in the same spinal cord slice preparation (Nerandzic et al., 2018).

Endocannabinoid-induced CB_1_ receptor activity has been associated with the synthesis of the 2-arachidonoylglycerol (2-AG), which in the spinal dorsal horn is proposed to be synthesized by DAGLα-expressing postsynaptic neurons under the control of the activity of the metabotropic glutamate receptor mGluR5 (Katona et al., 2006; Nyilas et al., 2009). 2-AG then diffuses to presynaptic terminals and activates CB_1_ receptors expressed on primary afferent endings (Ahluwalia et al., 2003; Nyilas et al., 2009; Veress et al., 2013). Carrageenan-induced paw inflammation does not induce changes in spinal 2-AG levels (Woodhams et al., 2012). Therefore, the loss of the CB_1_ receptor activation-induced inhibitory effect in inflammatory conditions could be due to reduced availability of 2-AG at the CB_1_ receptor, reduced 2-AG - CB_1_ receptor interaction, or reduced CB_1_ receptor downstream signaling in presynaptic terminals.

Additionally to increasing the basal mEPSC frequency, the CB_1_ receptor antagonist also blocked the 20:4-NAPE-induced inhibitory effect on mEPSC frequency in naive slices. It indicates that during 20:4-NAPE application to naive slices, presynaptic CB_1_ receptors were activated, in addition to 2-AG, also by anandamide. In contrast to naive slices, the CB_1_ receptor antagonist did not affect the 20:4-NAPE-induced inhibitory effects in slices dissected from animals after carrageenan injection. Instead, the 20:4-NAPE-induced inhibitory effect on mEPSC frequency was blocked by inhibiting exclusively TRPV1. In comparison to the 20:4-NAPE-induced inhibitory effect on sEPSC frequency and primary afferent fibers stimulation evoked EPSC amplitude underlying mechanism involved activation of both receptors. The switch of the 20:4-NAPE application-induced inhibitory effect on mEPSC frequency is highly perplexing for at least two reasons. First, inhibitory transmission, as well as action potential generation/propagation, were blocked during recordings. Hence, the TRPV1-mediated inhibitory effect should be produced by TRPV1 expressed by the presynaptic terminal itself. Nevertheless, how can activation of a non-selective cationic channel reduce transmitter release?

TRPV1 activation, in addition to leading to depolarization and action potential generation, also results in inhibitory effects through TRPV1-mediated Ca^2+^ influx. Thus, TRPV1 activation has been shown to inhibit high- (H) and low- (L) voltage-activated Ca^2+^ channels (VACC) in DRG neurons (Wu et al., 2005, 2006; Comunanza et al., 2011). Inhibition of L-VACC leads to reduced excitability of neurons, including primary sensory neurons, and inhibition of H-VACC results in reduced transmitter release (Kim et al., 2001; Park and Luo, 2010). However, activation of L-VACC and stochastic opening of H-VACC could be an important trigger for spontaneous glutamate release (Ermolyuk et al., 2013). Localized Ca^2+^ influx through TRPV1 activates large-conductance calcium- and voltage-activated potassium (BK) channels, which form a complex with TRPV1 (Wu et al., 2013). This effect shifts the membrane potential toward the resting potential, reducing the probability of H-VACC activation and subsequent transmitter release. The third mechanism which may contribute to reducing mEPSC frequency through TRPV1 activation is the dephosphorylation of proteins of the neurotransmitter-releasing apparatus by the phosphatases calcineurin or calmodulin, which are activated by TRPV1-mediated Ca^2+^ influx (Nichols et al., 1994; Sihra et al., 1995). Thus, it seems plausible to propose that one or the combined effects of the above-discussed mechanisms resulted in the unexpected TRPV1-mediated inhibitory effect on transmitter release. Unexpected TRPV1 activation-mediated inhibitory effect of capsaicin application on nociceptive synaptic transmission was also demonstrated by the inhibition of dorsal root electrical stimulation evoked EPSC amplitude (Baccei et al., 2003).

The second highly perplexing question arising from the TRPV1-mediated inhibition of transmitter release is how anandamide, synthesized after 20:4-NAPE application, changes target following peripheral inflammation. As discussed, most enzymes implicated in anandamide synthesis have been found in primary sensory neurons (Varga et al., 2014). The expression pattern of those enzymes in primary sensory neurons suggests that cells may express multiple pathways (Varga et al., 2014). The caveolin-rich membrane fractions associated with the CB_1_ receptor, ~30% of TRPV1, a major proportion of anandamide, and the anandamide synthesizing enzyme NAPE-PLD suggests compartmentalization of anandamide signaling through the CB_1_ receptor and TRPV1 (Bari et al., 2008; Rimmerman et al., 2008; Storti et al., 2015). Based on these findings, we propose that signaling, in primary sensory neurons, after inflammation of peripheral tissues, acts as a switch between TRPV1- and CB_1_ receptor-containing compartments where anandamide is synthesized. The presence of multiple anandamide-synthesizing enzymatic pathways in primary sensory neurons (Varga et al., 2014) suggests that the switch may involve the activation of different pathways. The spread of activity in the dorsal horn could constitute one of the modulators of that switch because, as we have reported previously, the 20:4-NAPE application-induced reduction in sEPSC frequency is mediated both by TRPV1 and the CB_1_ receptor (Nerandzic et al., 2018). Interestingly, the TRPV1-mediated Ca^2+^ influx-induced inhibitory effects have also been linked to microdomains (Comunanza et al., 2011). Those links raise the possibility of the presence of major molecular complexes formed by TRPV1, anandamide-synthesizing enzymes, BK channels, and VACC in primary sensory neurons. These could be present in addition to the TRPV1-CB_1_ receptor complexes we have demonstrated in primary sensory neurons (Chen et al., 2016). Taken together, these data suggest that a proportion of TRPV1 is activated by anandamide that is synthesized in close proximity to the ion channel. Anandamide synthesis is triggered by Ca^2+^ as well as activating protein kinases A and C (van der Stelt et al., 2005; Vellani et al., 2008). Importantly, TRPV1 activation also induces anandamide synthesis in primary sensory neurons (Nagy I. et al., 2009), and it is tempting to speculate that the capsaicin-induced anandamide synthesis might occur in TRPV1-NAPE-PLD-containing microdomains. Nevertheless, our data together suggest that a group of TRPV1 channels may act as a brake on spinal nociceptive processing by reducing the transmitter release in inflammation of peripheral tissues.

In summary, our results indicate an essential role of the anandamide precursor 20:4-NAPE and its converting enzyme NAPE-PLD in the modulation of spinal nociceptive signaling in the spinal terminals of primary sensory neurons. Further, our findings suggest that the switch of activation of TRPV1 and the CB_1_ receptor by endogenous anandamide in the spinal dorsal horn after peripheral inflammation significantly shapes spinal nociceptive processing hence pathological pain development. Our present results also highlight the complexity of the endovanilloid/endocannabinoid system in primary sensory neurons, which requires further investigation. Nevertheless, the data we present here provide further evidence for the significance of the endovanilloid/endocannabinoid system in pain modulation in primary sensory neurons.

## Data availability statement

The original contributions presented in the study are included in the article/supplementary material, further inquiries can be directed to the corresponding author/s.

## Ethics statement

The animal study was reviewed and approved by local Institutional Animal Care and Use Committee Institute of Physiology CAS.

## Author contributions

JP designed and supervised the study. VN, MP, AB, and DM conducted the experiments. VN, DS, and DM performed the data analysis. IN, DS, VN, and JP participated in writing the manuscript. All authors contributed to the article and approved the submitted version of the manuscript.

## Funding

This work was supported by the Grant Agency of the Czech Republic GACR 20-19136S, project LX22NPO5104 - funded by the European Union Next Generation EU and Institutional support RVO67985823.

## Conflict of interest

The authors declare that the research was conducted in the absence of any commercial or financial relationships that could be construed as a potential conflict of interest.

## Publisher’s note

All claims expressed in this article are solely those of the authors and do not necessarily represent those of their affiliated organizations, or those of the publisher, the editors and the reviewers. Any product that may be evaluated in this article, or claim that may be made by its manufacturer, is not guaranteed or endorsed by the publisher.

## Abbreviations

AEA: anandamide
CB: cannabinoid
DRG: dorsal root ganglion
mEPSC: miniature excitatory postsynaptic current
NAPE: N-arachidonoylphosphatidylethanolamine
NAPE-PLD: N-acyl phosphatidylethanolamine phospholipase D
PWL: paw withdrawal latency
TRPV1: transient receptor potential ion channel, vanilloid subfamily, type 1
TTX: tetrodotoxin
VACC: voltage-activated Ca^2+^ channels
